# Applying Genetic Risk Score to Improve Risk Assessment of New‐Onset Lupus Nephritis in Systemic Lupus Erythematosus: A Large‐Scale Prospective Cohort Study

**DOI:** 10.1002/mco2.70453

**Published:** 2025-11-14

**Authors:** Yufang Ding, Mucong Li, Wei Bai, Junyan Qian, Mengzhuo Cao, Jian Xu, Xinwang Duan, Hui Luo, Cheng Zhao, Feng Zhan, Min Yang, Rui Wu, Lijun Wu, Zhen Chen, Wei Wei, Yang Xu, Shangzhu Zhang, Xiaomei Leng, Qian Wang, Xinping Tian, Pei Gao, Xiaofeng Zeng, Xinzhuang Yang, Mengtao Li, Jiuliang Zhao

**Affiliations:** ^1^ Department of Rheumatology and Clinical Immunology Peking Union Medical College Hospital Chinese Academy of Medical Sciences Beijing China; ^2^ National Clinical Research Center for Dermatologic and Immunologic Diseases (NCRC‐DID) Ministry of Science & Technology Beijing China; ^3^ Key Laboratory of Rheumatology and Clinical Immunology Ministry of Education Beijing China; ^4^ Department of Rheumatology and Immunology First Affiliated Hospital of Kunming Medical University Kunming China; ^5^ Department of Rheumatology The Second Affiliated Hospital of Nanchang University Nanchang China; ^6^ Department of Rheumatology and Immunology Second Xiangya Hospital of Central South University Changsha China; ^7^ Department of Rheumatology and Immunology The People's Hospital of Guangxi Zhuang Autonomous Region Nanning China; ^8^ Department of Rheumatology and Immunology Hainan General Hospital Haikou China; ^9^ Department of Rheumatic & TCM Medical Center Nanfang Hospital Southern Medical University Guangzhou China; ^10^ Department of Rheumatology The First Affiliated Hospital of Nanchang University Nanchang China; ^11^ Department of Rheumatology and Immunology People's Hospital of Xinjiang Uygur Autonomous Region Urumqi China; ^12^ Department of Rheumatology The Second Affiliated Hospital of Fujian Medical University Quanzhou China; ^13^ Department of Rheumatology and Immunology Tianjin Medical University General Hospital Tianjin China; ^14^ Peking University Clinical Research Institute, Peking University First Hospital Beijing China; ^15^ Department of Medical Epidemiology and Biostatistics Karolinska Institutet Stockholm Sweden; ^16^ Department of Epidemiology and Biostatistics School of Public Health Peking University Beijing China; ^17^ Center For bioinformatics, National Infrastructures for Translational Medicine Institute of Clinical Medicine & Peking Union Medical College Hospital, Chinese Academy of Medical Sciences and Peking Union Medical College Beijing China

**Keywords:** genetic risk score, lupus nephritis, risk factors, systemic lupus erythematosus

## Abstract

Lupus nephritis (LN) is one of the most severe manifestation of systemic lupus erythematosus (SLE). However, reliable tools for predicting LN risk remain limited. In this multicenter prospective cohort study, we developed, validated, and refined a risk stratification model for new‐onset LN. A total of 2441 SLE patients without baseline renal involvement were consecutively enrolled from the Chinese SLE Treatment and Research (CSTAR) registry, with 215 (8.8%) developed LN in a median follow‐up time of 3.5 years. A combination of clinical predictors, age < 30 years, absence of arthritis, serositis, hypocomplementemia, and positive anti‐double‐stranded DNA antibodies identified a clinical high‐risk group (*n* = 537, 22.0%) with a 3‐year LN incidence of 18.1%. The model was externally validated in 451 patients, with 50 developed LN in a median follow‐up time of 3.4 years. In these patients, a genetic risk score (GRS) derived from 112 SLE‐associated loci was found to be independently associated with LN (HR = 3.19, 95% CI, 1.83–5.55, *p* = 4.36 × 10^−5^). Among clinically low‐risk individuals, those in the highest GRS quartiles (81/451, 18.0%) showed elevated 3‐year LN incidence (15.5% vs. 1.4%). New‐onset LN may be predicted using a combination of clinical risk factors, and integrating GRS further improves risk stratification, enabling early identification of high‐risk patients.

## Graphical abstract

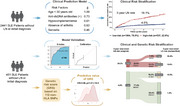

## 1 Introduction

Systemic lupus erythematosus (SLE) is a heterogeneous autoimmune disease characterized by diverse clinical features and fluctuating disease activity. Its pathogenesis involved autoantibodies production, chronic inflammation, and loss of immune tolerance, leading to severe tissue and organ damage. SLE can affect almost all organ systems, including skin and mucosa, kidney, nervous system, hematological system, and immune system. Among these, lupus nephritis (LN) is one of the most common and life‐threatening complications that occurs in approximately 40% of SLE patients [1] during the disease course. It is associated with considerable mortality [2] and is one of the most common causes of death in this population. The onset of LN exhibits substantial heterogeneity. While some patients develop nephritis concurrently with the initial diagnosis of SLE, others progress to renal involvement over months or years, often in the absence of early symptoms or laboratory abnormalities. This unpredictable course complicates early diagnosis and timely intervention.

Over the past few decades, significant advancements have been made in understanding the pathogenesis and therapeutic strategy for LN. Mechanistically, LN is driven by immune complex formation and deposition, elevated levels of pro‐inflammatory cytokines, activation of the complement pathway, and persistent immune cells activation [3]. These immunological abnormalities often precede the clinical diagnosis of LN, suggesting a subclinical phase during which early detection and intervention may alter disease trajectory. Based on its immune pathogenesis, LN is typically treated with glucocorticoids, hydroxychloroquine, and immunosuppressive therapy [4]. Several lines of evidence have demonstrated that early diagnosis and prompt initiation of immunosuppressive therapy may prevent the development of irreversible damage in SLE and may delay progression to chronic kidney disease in LN [5, 6]. Therefore, a great effort has been made to identify factors associated with developing LN in SLE patients [7].

In clinical practice, however, predicting which patients with SLE will progress to LN remains challenging. Various immune‐related molecules and renal function parameters are considered as potential predictors of LN. For instance, male sex, younger age at disease onset, serologic activity at SLE diagnosis, and positive anti‐complement 1q antibodies have been strongly linked to a higher risk of LN development [8, 9]. In a recently published study that included 570 patients without renal involvement at baseline, male, age under 26 years old, high‐titer anti‐double‐stranded DNA (anti‐dsDNA) antibodies, and low complement level were used to predict LN development [10]. Despite these insights, tools that could integrate these factors and facilitate the monitoring of LN development is still lacking in clinical practice. More importantly, most available tools focus on LN prognosis, rather than early risk assessment and onset prediction. Therefore, the absence of an accurate prediction model limits risk‐based monitoring and personalized treatment planning, and has sparked significant research interest.

Such evidence has shown that genetic susceptibility, environmental risk factors, and innate and adaptive immunity disorders play pivotal roles in the pathogenic mechanisms leading to SLE [11]. Over 100 single nucleotide polymorphisms (SNPs) associated with risk for SLE have been found. In the specific case of LN, several susceptibility genes, including those related to immune response and inflammation have been highlighted in its pathogenesis and progression, such as IRF5, TNIP1, STAT4, TNFSF4, APOL1, and PDGFRA [12]. Since SLE is a complex disease, individual genetic variants are generally considered to exert modest effects. Therefore, the genetic risk score (GRS), which aggregates the cumulative contribution of numerous variants, has emerged as a promising tool for predicting disease development and enabling prognostic stratification [13, 14]. In SLE, the association between cumulative genetic risk and disease manifestations has been reported [15, 16, 17, 18, 19]. Unlike serologic markers that may fluctuate with disease activity, genetic variants provide stable, lifelong information about disease risk. It is an attractive instrument for risk stratification that may provide information about disease predisposition from an asymptomatic stage.

Despite these advances, few studies have evaluated the utility of combining genetic and clinical information to predict new‐onset LN in SLE patients without LN at baseline. Furthermore, the added value of GRS for the precise prediction of new‐onset LN has not been fully elucidated. A comprehensive risk stratification model that incorporates both clinical risk factors and genetic predisposition could enable more precise, individualized management, guiding surveillance intensity and informing therapeutic decisions. Therefore, the objective of this study was to develop, validate, and refine a risk stratification tool for new‐onset LN in a large, multicenter, prospective SLE cohort. By integrating clinical parameters with GRS, we aimed to improve risk discrimination and provide a practical tool for early identification of patients at high risk for LN, ultimately contributing to more personalized and effective disease management.

## 2 Results

### 2.1 Study Population

**FIGURE 1:**
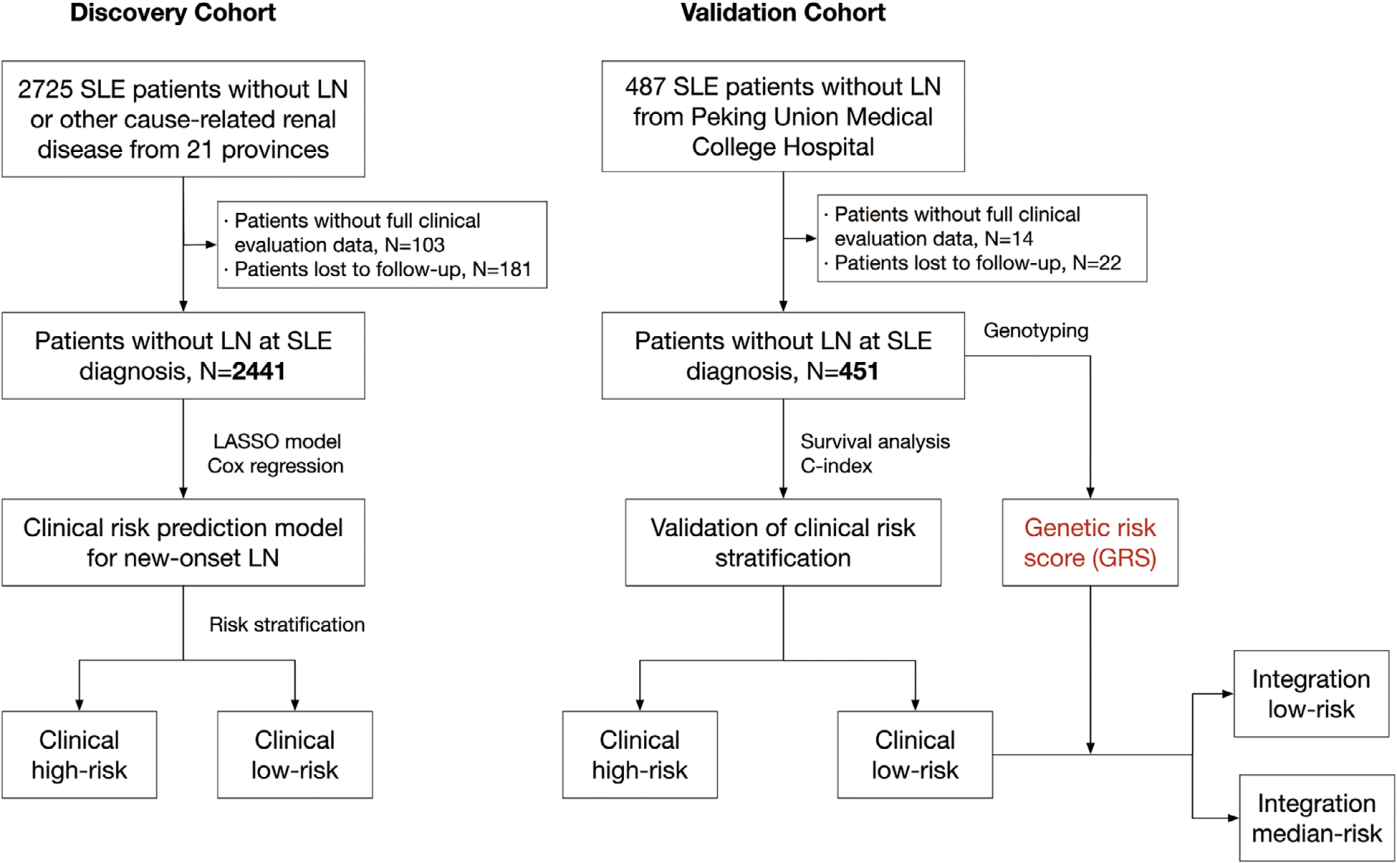
Flowchart of the study.

We consecutively collected 2725 SLE without LN or other cause‐related renal diseases diagnosed from January 2010 to June 2022 from 21 provinces (Figure 1). After excluding 103 with incomplete core clinical evaluation data (3.8%) and 181 patients lost to follow‐up (6.6%), we assessed 2441 patients as the discovery cohort (Table 1). Among these patients, 93.7% (2287/2441) were women, with a mean age at diagnosis of 34.9 ± 11.4 years old and a mean disease duration of 1.8 ± 1.2 years. Note that 1262 (51.7%) showed positive anti‐dsDNA antibodies at diagnosis, and 1130 (46.3%) showed hypocomplementemia. Patients were followed up for a median follow‐up time of 3.7 years (the longest follow‐up was 11.4 years), totaling 11, 349 study visits. Furthermore, we independently enrolled 451 patients from one of the centers included in our study (Peking Union Medical College Hospital) to validate the utility of clinical risk factors and assess the association between genetic risk and new‐onset LN (Table 1). Patients in both the discovery and validation cohorts hailed from the same geographical background. There was no significant difference in the majority of clinical and laboratory features between the two cohorts.

**TABLE 1:** Clinical manifestations, laboratory features, and medications at baseline in the discovery and the validation cohort.

	Discovery cohort		Validation cohort	
Characteristic	All patients (*n* = 2441)	Developed LN (*n* = 215)	All patients (*n* = 451)	Developed LN (*n* = 50)
Female, *n* (%)	2287 (93.7)	201 (93.5)	414 (91.8)	48 (96.0)
Age at diagnosis (years), mean ± SD	34.9 ± 11.4	35.6 ± 11.5	33.9 ± 9.4	27.5 ± 6.6
Disease duration (years), mean ± SD	1.8 ± 1.2	1.7 ± 0.9	1.7 ± 1.2	1.8 ± 1.4
Follow up (years), median (interquartile range)	3.7 (2.6, 5.3)	3.5 (2.0, 5.0)	3.9(2.0, 5.7)	3.4 (1.4, 5.5)
Malar rash, *n* (%)	999 (40.9)	75 (34.9)	203 (45.0)	21 (42.0)
Discoid lesions, *n* (%)	305 (12.5)	28 (13.0)	36 (8.0)	6 (12.0)
Arthritis, *n* (%)	1316 (53.9)	76 (35.3)	246 (54.5)	18 (36.0)
Oral ulcerations, *n* (%)	462 (18.9)	43 (20.0)	106 (23.5)	12 (24.0)
Serositis, *n* (%)	272 (11.1)	43 (20.0)	56 (12.4)	15 (30.0)
Alopecia, *n* (%)	934 (38.3)	70 (32.6)	170 (37.7)	17 (34.0)
Hematological involvement, *n* (%)	1349 (55.3)	126 (58.6)	181 (40.1)	32 (64.0)
Neurological involvement, *n* (%)	157 (6.4)	13 (6.0)	16 (3.5)	2 (4.0)
Hypocomplementemia, *n* (%)	1130 (46.3)	161 (74.9)	204 (45.2)	35 (70.0)
Anti‐nuclear antibodies (ANA), *n* (%)	2441 (100.0)	215 (100.0)	451 (100.0)	50 (100.0)
Anti‐double stranded DNA (anti‐dsDNA) antibodies, *n* (%)	1262 (51.7)	159 (74.0)	227 (50.3)	37 (74.0)
Anti‐Sm antibodies, *n* (%)	783 (32.1)	102 (47.4)	161 (35.7)	28 (56.0)
Anti‐RNP antibodies, *n* (%)	754 (30.9)	99 (46.0)	150 (33.3)	25(50.0)
Anti‐SSA antibodies, *n* (%)	1385 (56.7)	116 (54.0)	279 (61.9)	29 (58.0)
Anti‐SSB antibodies, *n* (%)	513 (21.0)	41 (19.1)	100 (22.2)	10 (20.0)
Anti‐RibP antibodies, *n* (%)	613 (25.1)	73 (34.0)	122 (27.1)	19 (38.0)
Anti‐nucleosome antibodies, *n* (%)	494 (20.2)	119 (55.3)	94 (20.8)	15 (30.0)
Anti‐histone antibodies, *n* (%)	388 (15.9)	65 (30.2)	80 (17.7)	20 (40.0)
Anti‐phospholipid antibodies, *n* (%)	798 (32.7)	116 (54.0)	167 (37.0)	41 (82.0)
SLEDAI‐2K, mean ± SD	5.1 ± 3.8	6.6 ± 3.3	4.8 ± 3.1	6.6 ± 3.3
Treatment^a^				
Glucocorticoid usage, *n* (%)	2292 (93.9)	202 (94.0)	435 (96.5)	48 (96.0)
Hydroxychloroquine (HCQ), *n* (%)	2118 (86.8)	181 (84.2)	401 (88.9)	45 (90.0)
Immunosuppressive therapy^b^, *n* (%)	654 (26.8)	56 (26.0)	85 (18.8)	13 (26.0)

^a^ Anti‐Sm antibodies, anti‐Smith (Sm) antibodies; Anti‐RNP antibodies, anti‐ribonucleoprotein (RNP) antibodies; Anti‐SSA antibodies, anti‐Sjogren Syndrome A (anti‐SSA) antibodies; Anti‐SSB antibodies, anti‐Sjogren Syndrome B (anti‐SSB) antibodies; Anti‐RibP antibodies, anti‐ribosomal P protein (anti‐RibP) antibodies; SLEDAI‐2K, Systemic Lupus Erythematosus Disease Activity Index 2000 (SLEDAI‐2K).

^b^ Medications being used at SLE diagnosis (baseline).

^c^ Immunosuppressive therapy included cyclophosphamide, methotrexate, mycophenolate mofetil, azathioprine, calcineurin inhibitors, and (or) azathioprine.

### 2.2 New‐onset LN

In the discovery cohort, the overall incidence of new‐onset LN was 8.8% (215/2441) within a median (interquartile range [IQR]) follow‐up time of 3.5 (2.0, 5.0) years (Figure S1A). The cumulative event occurrence incidence at 1, 3, and 5 were 3.2%, 7.6%, and 10.5%, respectively. Among patients who developed LN during follow‐up, 52.1% (112/215) were diagnosed histologically. LN was classified as class III (*n* = 21), class IV (*n* = 43), class V (*n* = 17), mixed class (*n* = 31) (20 patients were in class III+V and 11 patients were in class IV+V) based on the International Society of Nephrology/Renal Pathology Society criteria [20].

### 2.3 The clinical risk stratification for new‐onset LN

To identify clinical risk factors associated with new‐onset LN, we employed the Least Absolute Shrinkage and Selection Operator (LASSO) regression model and Cox regression analysis. Five variables with nonzero coefficients were selected from 18 candidate predictors via 1 SE criteria (Figure S2). Then, the five predictors were further involved in multivariable Cox regression after testing for the proportional hazards assumption (Table 2): age <30 years old (hazard ratio [HR] = 2.99, 95% confidence interval [CI], 2.25–3.97, *p* = 3.53 × 10^−14^), absence of arthritis at SLE diagnosis (HR = 1.84, 95% CI, 1.33–2.53, *p* = 1.29 × 10^−5^), serositis (HR = 1.58, 95% CI, 1.12–2.22, *p* = 0.009), hypocomplementemia (HR = 1.89, 95% CI, 1.42–2.51, *p* = 1.94 × 10^−4^), and positive anti‐dsDNA antibodies (HR = 2.08, 95% CI, 1.51–2.89, *p* = 9.84 × 10^−6^). The integration of the five risk factors provided an incremental area under the time‐dependent receiver operating characteristics (ROC) curve (AUC) of 0.792 (95% CI, 0.761–0.823).

**TABLE 2:** Multivariable Cox regression analysis revealed clinical risk factors for new‐onset LN in the discovery and validation cohort.

	Discovery cohort				Validation cohort			
Predictor variables	*β*	HR	95% CI	*p* value	*β*	HR	95% CI	*p* value
Age <30 years old	1.09	2.99	2.25–3.97	3.53 × 10^−14^	1.36	3.90	2.03–7.50	4.54 × 10^−5^
Absence of arthritis	0.63	1.89	1.42–2.51	1.29 × 10^−5^	0.52	1.68	1.09–3.11	0.047
Serositis	0.46	1.58	1.12–2.22	0.009	0.93	2.53	1.32–4.82	0.005
Hypocomplementemia	0.61	1.84	1.33–2.53	1.94 × 10^−4^	0.63	1.87	1.01–3.55	0.035
Anti‐dsDNA antibodies	0.73	2.08	1.51–2.89	9.84 × 10^−6^	0.76	2.14	1.12–4.10	0.022

**FIGURE 2:**
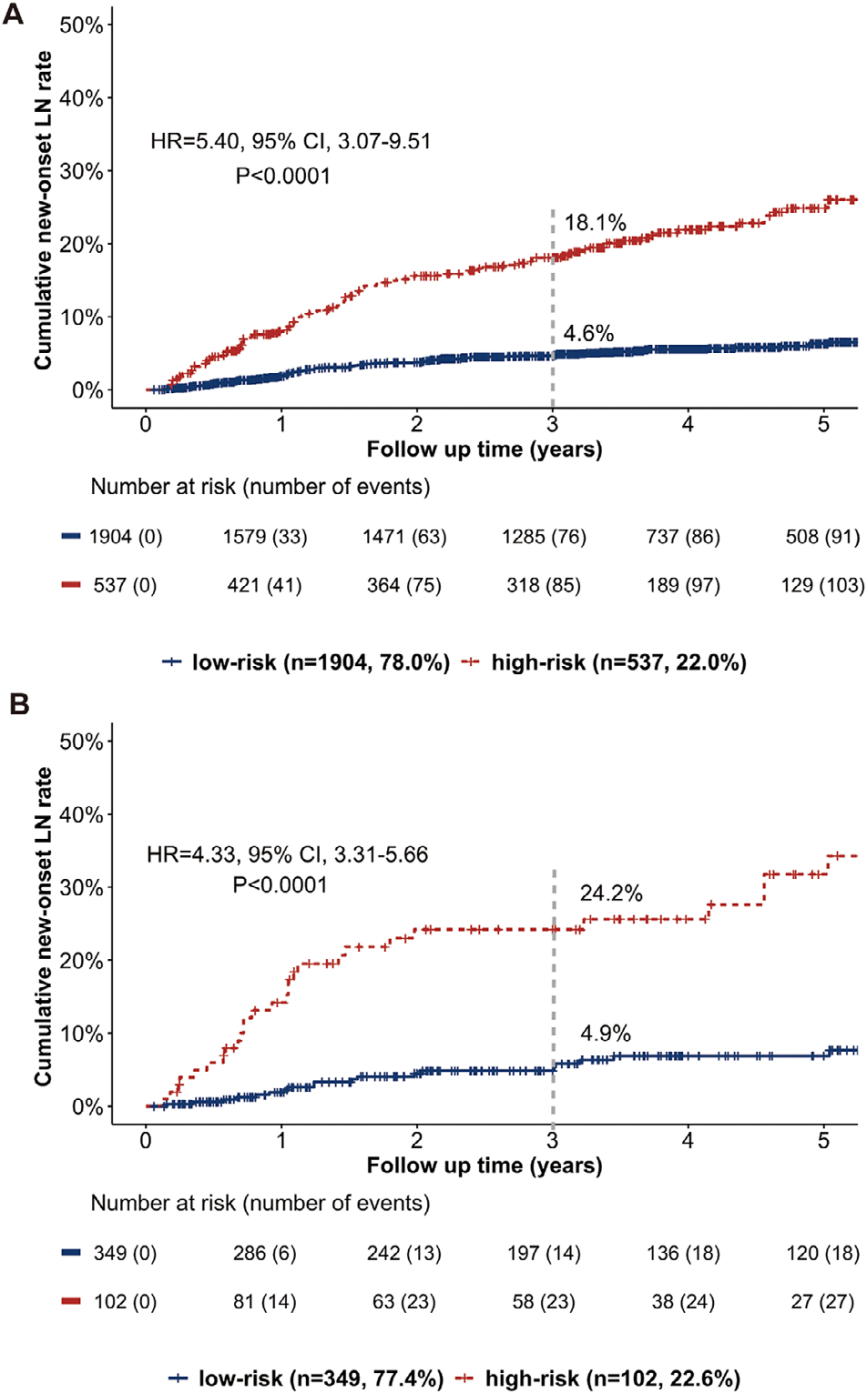
New‐onset LN risk stratification based on clinical risk factors in the (A) discovery cohort and the (B) validation cohort. In the discovery cohort, 537 (22.0%) patients were classified into the clinical high‐risk group, among which 85 patients (18.1%) developed LN during follow‐up within 3 years. Note that 1904 (78.0%) patients were classified into the clinical low‐risk group, with a 3‐year cumulative incidence of LN of 4.6%. In the validation cohort, 102 (22.6%) were classified into the clinical high‐risk group with a 3‐year cumulative LN incidence of 24.2%; 349 (77.4%) were classified into the clinical low‐risk group, with a 3‐year cumulative LN incidence of 4.9%.

Patients with at least four clinical risk factors, or those younger than 30 with two or more additional risk factors, were defined as the clinical high‐risk group. Based on this criterion, 537 (22.0%) patients were classified into this group. The 3‐year cumulative incidence of LN was 18.1% in the high‐risk group. For the clinical low‐risk group, the 3‐year cumulative incidence of LN was 4.6% (Figure 2A).

### 2.4 Validation of Clinical Risk Stratification

We further enrolled another 451 patients to validate the predictive value of the clinical risk factors. During a median (IQR) follow‐up time of 3.4 (1.4, 5.5) years, 50 patients (11.1%) developed LN (Figure S1B) and 36 patients were confirmed by renal biopsy. Among the 36 patients, 30 were proliferative LN (class III or IV with or without concurrent class V disease). Similar to that in the discovery cohort, multivariable Cox regression was used to assess the association between clinical risk factors and LN development. Consistently, the five traditional clinical risk factors also revealed a positive correlation with LN in the validation cohort (Table 2): age <30 years old (HR = 3.90, 95% CI, 2.03–7.50, *p* = 4.54 × 10^−5^), absence of arthritis at SLE diagnosis (HR = 1.68, 95% CI, 1.09–3.11, *p* = 0.047), serositis (HR = 2.53, 95% CI, 1.32–4.82, *p* = 0.005), hypocomplementemia (HR = 1.87, 95% CI, 1.01–3.55, *p* = 0.035), and positive anti‐dsDNA antibodies (HR = 2.14, 95% CI, 1.12–4.10, *p* = 0.022).

Of the 451 patients in the validatio cohort, 102 (22.6%) were classified into clinical high‐risk group with a 3‐year cumulative LN incidence of 24.2%, while 349 (77.4%) were in low‐risk group with a 3‐year LN cumulative LN incidence of 4.9% (Table 3, Figure 2B).

**TABLE 3:** The cumulative incidence of LN at 1, 3, and 5 years based on clinical‐risk stratification and the integration risk stratification in the 451 SLE patients with genotyping data.

	Clinical risk stratification		Clinical and genetic risk stratification		
	Low risk (*N* = 349, 77.4%)	High risk (*N* = 102, 22.6%)	Low risk^a^ (*N* = 268, 59.4%)	Medium risk^b^ (*N* = 81, 18.0%)	High risk (*N* = 102, 22.6%)
1‐year	1.9%	14.2%	0.4%	6.6%	14.2%
3‐years	4.9%	24.2%	1.4%	15.5%	24.2%
5‐years	6.9%	31.8%	2.1%	21.1%	31.8%

^a^ Clinical low‐risk group with low‐ or median‐GRS quartiles.

^b^ Clinical low‐risk group with high‐GRS quartiles.

### 2.5 Genetic Risk Score Was an Independent Predictor of LN Development

Genotyping was conducted for 451 patients to assess the impact of genetic risk on the development of LN. A weighted GRS was performed based on 112 SNPs associated with SLE [21] and was analyzed as a continuous variable in regression analyses. The GRS was categorized into extreme quartiles for risk stratification analysis. Specifically, 113 patients were in high‐GRS quartiles, 225 were in median‐GRS quartiles, and 113 were in low‐GRS quartiles. The median (IQR) of GRS was 7.8 (7.5, 8.1). Figure S3 illustrates the distribution density of the continuous GRS score, with a mean of 8.42 ± 0.40 in LN patients and 7.89 ± 0.41 in those patients without LN during the follow‐up period (*p* < 0.001). The mean number of risk alleles was 61.30 ± 4.53 in LN patients, compared to 55.35 ± 4.71 in those without LN (*p* < 0.001).

To further elucidate the association between the risk of LN and individual GRS, Pearson correlation analysis and the multivariable Cox regression model were conducted. The correlation analysis demonstrated a significant rise in the incidence of new‐onset LN in tandem with rising GRS values (*p* = 1.06 × 10^−4^). The Cox regression analysis confirmed a positive correlation between GRS and the risk of LN (HR = 3.98, 95% CI, 2.12–7.44, *p* = 1.61 × 10^−5^), after adjusting for onset age, sex, and disease duration. The ability of GRS to discriminate between SLE patients with and without new‐onset LN was moderate (AUC = 0.667). To analyze the independent effect of genetic risk load on new‐onset LN, we performed a multivariable Cox regression. After adjusting for the five clinical risk factors, the adjusted HR was 3.19 (95% CI, 1.83–5.55, *p* = 4.36 × 10^−5^), indicating the independent predictive value of GRS. In addition, we explored the association between GRS and LN development across different age groups at SLE onset. Within our cohort, the prediction accuracy of the GRS was higher in patients aged <30 years at SLE onset (HR = 4.19, *p* = 0.031) compared with those aged ≥ 30 years (HR = 3.25, *p* = 0.001). Given that proliferative LN is an important factor in LN prognosis, we analyzed LN‐free survival until the development of proliferative LN separately. In biopsy‐confirmed LN, the relationship between proliferative LN and GRS was consistent (HR = 4.72, 95% CI, 3.18–8.05, *p* = 4.18 × 10^−7^).

Subsequently, we compared the highest and lowest GRS quartiles and found that SLE patients in the top GRS quartile were at a more than a 3.89‐fold and 2.61‐fold higher risk of developing LN compared to those in the median or bottom GRS quartile, respectively. Compared with the low‐GRS quartile, patients in the high‐GRS quartiles showed 14.2% higher incidence of developing LN (20.4% vs. 6.2%, *p* < 0.001). We then evaluated the incidence rate of LN at 1, 3, and 5 years based on GRS quartiles. As shown in Table S1, the 3‐year cumulative incidence of LN in low‐, median‐, and high‐GRS quartiles was 3.0%, 8.7%, and 17.4%, respectively.

### 2.6 Improving Risk Stratification Through the Integration of GRS

To assess the combined effect of the GRS and clinical risk factors on the risk of LN, we performed time‐dependent ROC analysis and DeLong's test. The risk classification of new‐onset LN based on the five traditional clinical risk factors was found to have an AUC of 0.789 (95% CI, 0.764–0.813). The integration of GRS, age <30 years, absence of arthritis, serositis, hypocomplementemia, and positive anti‐dsDNA antibodies provided an incremental AUC of 0.842 (95% CI, 0.829–0.873) and the optimism‐corrected AUC was 0.838 (95% CI, 0.823–0.897) in predicting LN development within 3 years. The integration of GRS and the five clinical risk factors showed better predictive ability (DeLong's test, *p* < 0.001). These results highlight the added value of genetic information in LN prediction.

**FIGURE 3:**
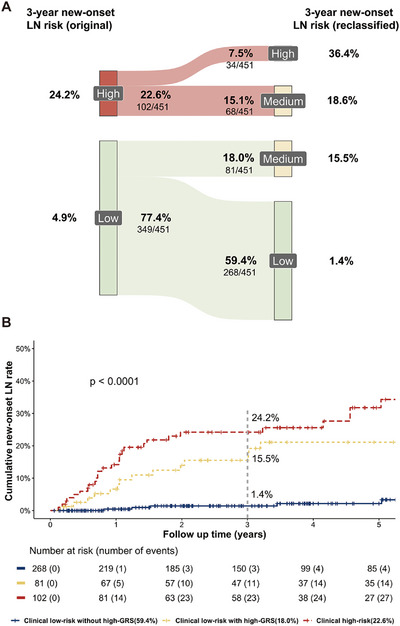
(A) Sankey diagrams visualizing reclassification by GRS. The figure shows the subgroups which were genetically upgraded (high GRS quartiles) from a lower risk category in clinical risk stratification to a higher risk category in the integration risk stratification (clinical low risk to integration low or medium risk, clinical high‐risk to integration medium or high risk); (B) Kaplan–Meier analysis of different risk grades of new‐onset LN proportion based on the integration of GRS and clinical risk stratification.

Furthermore, the combination of GRS and clinical risk factors proved to be an effective tool for risk stratification in LN. As shown in the Sankey diagram (Figure 3A), after incorporating genetic factor, patients in both the clinically low‐ and high‐risk group could be further stratified. The 3‐year cumulative incidence of LN in the clinical high‐risk group with high‐GRS quartiles (34/451, 7.5%), clinical high‐risk group with low‐ or median‐GRS quartiles (68/451, 15.1%), clinical low‐risk group with high‐GRS quartiles (81/451, 18.0%), and clinical low‐risk group with low‐ or median‐GRS quartiles (268/451, 59.4%) were 36.4%, 18.6%, 15.5%, and 1.4%, respectively (Figure 3B). These results suggest that the genetic risk factor is indispensable for LN risk stratification.

## 3 Discussion

LN is a common and potentially fatal complication of SLE patients. Accurately predicting which patients are likely to develop LN at the time of SLE diagnosis is vital for optimizing treatment decisions. Previous studies have assessed the predictive value of various risk factors and genetic susceptibility loci [22] for LN. However, an effective and clinically practical method to predict LN in lupus patients is still in urgently needed. Based on our large prospective cohort, we developed a set of genetic and clinical risk factors to generate individualized risk estimates for LN in SLE patients without baseline renal involvement.

Our study demonstrated that patients younger than 30 years old, serositis, without arthritis, with hypocomplementemia, or with positive anti‐dsDNA antibodies at SLE diagnosis showed a higher risk of subsequent LN development. Previous studies have proposed anti‐dsDNA antibodies as a biomarker for LN and renal relapse. Anti‐dsDNA antibodies target the double‐strands DNA in the chromatin [23]. They can bind directly to renal cells via antigens or indirectly through chromatin fragments deposited on the cell surface. Anti‐dsDNA antibodies are thought to be involved in the inflammatory pathways [24] and may induce cell apoptosis [25]. They can colocalize with nucleosomes in the glomerular basement membrane (GBM) [26]. GBM cells undergo early intraglomerular apoptosis in LN, which can lead to the abnormal exposure of chromatin fragments. The nucleosomes released by dying cells become immunogenic and stimulated autoreactive B cells to secrete anti‐dsDNA and anti‐nucleosome antibodies. The binding of those autoantibodies to the deposited antigen in the GBM contributes to the development of glomerulopathy [27]. Besides anti‐dsDNA antibodies, complement activation is also a crucial event in the pathogenesis of LN. Decreased C3 and C4 levels have been observed in about 75% of SLE patients with focal nephritis and 90% in those with diffuse nephritis [28]. The deposition of complement proteins in the glomeruli is likely a key feature of LN [29]. Both complement split products and cell‐bound complement activation products can monitor LN in SLE patients [30]. Another factor identified in our study was arthritis, which appeared to serve as a protective factor against new‐onset LN. An international inception cohort suggests that LN patients show a lower frequency of joint or cutaneous involvement [31]. Further studies are needed to support and elucidate the relationship between arthritis and LN.

Importantly, our study is the first to confirm the independent role of GRS in new‐onset LN prediction and its additive value in LN risk stratification. It has been suggested that the GRS is an effective tool for assessing the early diagnosis, disease manifestations, and prognosis of SLE. In the European population, it has been reported by Reis et al. [16] that high genetic risk, based on 57 SLE risk SNPs at genome‐wide significance, was associated with a higher prevalence of damage accrual (OR = 1.47, 95% CI, 1.06–2.04, *p* = 2.0 × 10^−2^), renal disorder (OR = 2.22, 95% CI, 1.50–3.27, *p* = 5.9 × 10^−5^), and decreased overall survival (HR = 1.83, 95% CI, 1.02–3.30, *p* = 4.3 × 10^−2^). Higher genetic risk was also correlated with positive anti‐dsDNA antibodies (OR = 1.83, 95% CI, 1.19–2.81, p = 6.1 × 10^−3^), a pivotal risk factor for new‐onset LN. Besides, in an East Asian cohort [19], a higher GRS was found to modulate the pathogenesis of proliferative and membranous LN (HR = 1.98 for class III or IV; HR = 2.79 for class V). In another retrospective case‐control study, Shin et al. also analyzed the association between non‐HLA GRS and the presence of LN [32]. They found that LN patients had higher GRSs (OR = 1.16, *p* = 0.012), and this relationship may be mediated by positive anti‐Sm antibodies and hypocomplementemia. Similar to the previous study, our study assessed the genetic risk using GRS based on 112 non‐HLA SLE susceptive loci in the East Asian population. Using a large, multicenter, prospective cohort of patients without LN at baseline, we provided compelling evidence that patients with high genetic risk had a significantly higher risk of new‐onset LN within 3 years (17.4% vs. 3.0%). GRS has been identified as a promising predictor of LN, independent of well‐established clinical risk factors such as anti‐dsDNA antibodies and hypocomplementemia. Besides, the integration of GRS and clinical risk factors can provide more accurate information in LN risk stratification, especially for patients classified as low‐risk based on clinical factors. In the clinical low‐risk group, patients in the high‐GRS quartiles exhibited significantly higher risk for LN than those without (15.5% vs. 1.4%). These findings underscore the potential value of GRS in the SLE personalized management.

Our study delved into the prediction of LN within a multi‐center cohort of well‐characterized SLE patients. This study has several limitations. First, not all patients underwent renal biopsy due to patients’ concerns about the invasiveness of the procedure in China. It is unknown how many patients with a clinical diagnosis of LN who did not receive a kidney biopsy may have had acute tubular necrosis, acute interstitial nephritis, renal thrombotic microangiopathy, renal antiphospholipid syndrome, or lupus podocytopathy, instead of immune complex LN. It is also unknown how many of the 2226 patients without a clinical diagnosis of LN and without a kidney biopsy would have shown histologic evidence of LN. To address this limitation, we conducted a comparison between patients with and without a kidney biopsy. We found no significant differences in demographic or baseline SLE characteristics, although patients who received biopsy generally exhibited more severe renal manifestations. Second, the risk factors discovered in our study may be more relevant to patients of similar race or disease stages as those in the study cohort. The applicability of the risk assessment based on GRS and clinical risk factors requires further validation in a racially and geographically diverse cohort. Additionally, given the observational nature of our study, causality between the risk stratification and early intervention strategies cannot be established. Interventional studies are needed to explore whether our risk assessment tool can guide management decisions and improve the prognosis in SLE.

## 4 Conclusion

Our study provides further evidence supporting the predictability of new‐onset LN. In a multicenter prospective cohort, we demonstrated that LN development can be effectively predicted using a combination of clinical risk factors, including younger age, serositis, absence of arthritis, hypocomplementemia, and anti‐dsDNA antibodies. More importantly, our study highlighted the value of genetic susceptibility to SLE, as measured by GRS, which is a promising tool for clinicians to predict and stratify LN risk. The GRS could further refine risk prediction within the clinical risk group, demonstrating a great potential to identify high‐risk individuals for monitoring in clinical utility. However, future endeavors are necessary to validate the accuracy and advantage of GRS in LN risk stratification.

## 5 Materials and Methods

### 5.1 Study Design

The study adheres to the Transparent Reporting of Strengthening the Reporting of Observational Studies in Epidemiology (STROBE) checklist [33] and the Performing Polygenic Risk Score Analyses Guideline [13, 34].

### 5.2 Study Participants and Data Collection

Patients diagnosed with SLE between January 2010 and June 2022 were consecutively enrolled from 21 provinces through the Chinese SLE treatment and research (CSTAR) online registry [35, 36], which had completed follow‐up quality control at the time of analysis. The inclusion criteria comprised all patients who (a) met the revised 1997 American College of Rheumatology (ACR) classification criteria for SLE [37], the 2012 Systemic Lupus International Collaborating Centers (SLICC) group classification criteria [38], or 2019 European League Against Rheumatism (EULAR)/ACR classification criteria [39], (b) without renal involvement at diagnosis (defined by negative urinary protein, or spot urine protein‐to‐creatinine ratio ≤ 500 mg/g, or proteinuria <0.5 g/24 h, with or without hematuria or cell casts). Patients with renal diseases likely caused by factors other than SLE, such as renal diseases associated with drug or plasma cell dyscrasias, were excluded. This study was approved by the Institute Review Board of Peking Union Medical College Hospital (Approval number, JS‐3386D, K‐5745). Other centers obtained ethical approval from the local institutional review board.

At SLE diagnosis and during each follow‐up, data on demographics, clinical characteristics, laboratory results, and treatment regimens were collected. Blood samples for genotyping were taken at baseline and stored at −80°C for further analysis. Demographic data included sex (male or female), age, and disease duration. Clinical features, including mucocutaneous involvement, arthritis, serositis, hematological involvement, and neuropsychiatric involvement, were documented, along with laboratory test results, such as serum creatinine, complement levels, autoantibodies, urinary sediment, and urine protein. Disease activity was assessed annually using Systemic Lupus Erythematosus Disease Activity Index 2000 (SLEDAI‐2K) and organ damage according to the SLICC/ACR Damage Index (SDI). Treatment regimens, including glucocorticoids, hydroxychloroquine, and immunosuppressive agents, were recorded at SLE diagnosis and during each visit.

### 5.3 Follow‐Up and Outcome Measures

All eligible patients were followed up every 3–6 months from the time of SLE diagnosis until developed LN or any censoring event (loss to follow‐up or study end date).

We defined the baseline as the time of SLE diagnosis and the endpoint as the occurrence of LN or the latest follow‐up. Renal parameters, monitored by serum creatinine, 24‐h proteinuria or urine protein‐to‐creatinine ratio, and urinary sediment, were assessed at baseline and during follow‐ups. LN was diagnosed either histologically (based on renal biopsy compatible with LN histopathology classes III, IV, and V) [40] or clinically (based on persistent proteinuria ≥ 0.5 g/24 h, or equivalent spot urine protein‐to‐creatinine ratio, or active urinary sediment) [38] assessed by rheumatologists and nephrologists.

### 5.4 Measures

In the risk factors analysis, candidate predictors for new‐onset LN included age, gender, SLE disease duration, clinical manifestations, and autoantibody profiles. The definitions of the 18 candidate predictors are provided in Table S2. The optimal age cut‐off was set based on the average age of Chinese LN patients [41]. The clinical manifestations included malar rash, discoid lesions, arthritis, oral ulcerations, alopecia, serositis, hypocomplementemia, neurological involvement, and hematological involvement. The autoantibody profiles included anti‐dsDNA, anti‐Smith (Sm), anti‐ribonucleoprotein (RNP), anti‐Sjogren Syndrome A (anti‐SSA), anti‐Sjogren Syndrome B (anti‐SSB), and anti‐ribosomal P protein (anti‐RibP). The autoantibodies were all measured at the lab of each center with the same assays. Anti‐nuclear antibodies (ANA) were detected by indirect immunofluorescence (IIF) assay with the Hep‐2 cell line. Anti‐dsDNA antibodies were assessed using the Crithidia luciliae immunofluorescence test (CLIFT) and enzyme‐linked immunosorbent assay (ELISA) (EA 1571–9601 G). Positive results were defined as ≥ 1:5 for CLIFT and ≥ 100 for ELISA as recommended by the manufacturer's guidelines. Patients were considered positive for anti‐dsDNA antibodies if either CLIFT or ELISA yielded a positive result. Hypocomplementemia was defined as low C3 and (or) C4 levels.

### 5.5 Genotyping and Imputation

The genotyping was completed by using the Illumina Infinium Asian Screening Array‐24 v1.0 (ASA) and the Illumina iScan System according to the manufacturer's instructions. The ASA chip contains more than 750, 000 markers. We conducted standard QC at both the variant level and sample level according to the literature. In brief, the variant should pass the following criteria: (1) Hardy–Weinberg equilibrium score > 10e‐6, (2) variant calling rate among all participants ≥ 0.9. We also excluded individuals from the association analysis who (1) were sex‐discordant/ambiguous; (2) had an excessive heterozygosity rate (> 3 standard deviations from the mean); (3) outlier samples in principal component analysis; (4) low sample call rate (≤ 0.9); (5) relatedness among individuals was calculated using the identity by descent (IBD) analysis. For each pair of individuals with PI_HAT > 0.2, we excluded the one with a lower call rate. Samples and variants passing the QC were then phased with shapeit (v2.r904) and imputed with minimac4 (V1.0.2), respectively, by using the default parameters and the 1000 genomes cosmopolitan panel phase 3 version 5 (1000G p3v5) as reference. Finally, common variants (MAF > 1%) passing basic quality control and with estimated imputation accuracy Rsq > 0.3 were included for further analysis.

### 5.6 Genetic Risk Score

SNPs for the GRS were identified from the largest meta‐analysis of East Asian SLE patients to date [21]. Weighted GRS were assigned to each individual based on 112 non‐human leukocyte antigen (HLA) genetic loci. For each susceptibility SNP, we multiplied the number of risk alleles in each individual and the natural odd ratio (OR) for SLE susceptibility based on weights derived from the meta‐analysis. The sum of all products for each patient who passes genotyping data quality control procedures was defined as the GRS.

### 5.7 Statistical Analysis

Descriptive statistics are presented as mean ± standard deviations (SD) or median (IQR) for continuous variables and as frequencies for categorical variables. The *t*‐test was used to compare continuous variables, while categorical data were analyzed using Pearson's chi‐square test.

First, the association between clinical risk factors and LN development was analyzed using the LASSO method [42] and Cox proportional hazards models. In the LASSO regression, the optimal model was identified by 20‐fold cross‐validation based on the 1 standard error of the minimum criteria. Variables with nonzero coefficients were subsequently included in the multivariable Cox regression analysis after testing for Cox proportional hazards assumption. HR and 95% CIs were estimated by fitting the Cox regression. A set of clinical risk factors was selected to define clinical risk categories based on predictive utility and clinical applicability.

Then, we conducted Pearson correlation analysis and multivariable analysis to examine the independent association between GRS and time‐to‐event outcome. The GRS was treated as a continuous variable in the association analysis and was categorized into extreme quartiles for risk stratification.

Additionally, the added value of GRS in clinical risk stratification was assessed. DeLong's test was performed for the statistical comparison of AUC. The Kaplan–Meier method was used to calculate the cumulative new‐onset LN rates during follow‐up. The log‐rank test was applied to compare different risk groups. *p* < 0.05 was considered statistically significant, and all statistical tests were two‐tailed probability tests. Statistical analysis was performed using R Statistical Software, version 3.6.1. (R Foundation for Statistical Computing, Vienna, Austria).

## Author Contributions

Y.F.D., M.C.L., W.B., J.Y.Q., J.X., M.Z.C., X.W.D., H.L., C.Z., F.Z., M.Y., R.W., L.J.W., Z.C., W.W., Y.X., S.Z.Z., X.M.L., Q.W., X.P.T., P.G., X.F.Z., X.Z.Y., M.T.L., and J.L.Z. contributed to the study conception, design, and data collection. Y.F.D., M.T.L., and J.L.Z. designed the study and had unrestricted access to all data reported in this study. Y.F.D. and X.Z.Y. prepared the material and performed the analysis. Y.F.D., X.Z.Y., M.T.L., and J.L.Z. conceived the project, led the study, and supervised the work. The first draft of the manuscript was written by Y.F.D. and J.L.Z. All authors accepted responsibility for the work. All authors have read and approved the final manuscript.

## Funding

This work was supported by the Chinese National Key Technology R&D Program, Ministry of Science and Technology (2021YFC2501300), National Natural Science Foundation of China (32441090), CAMS Innovation Fund for Medical Sciences (CIFMS) (2023‐I2M‐C&T‐B‐047), and National High Level Hospital Clinical Research Funding (2025‐PUMCH‐D‐001; 2022‐PUMCH‐B‐013,C‐002,D‐009).

## Ethics Statement

This study was approved by the institutional review board (IRB) of Peking Union Medical College Hospital (Approval number, JS‐3386D, K‐5745). Informed consent was obtained. Other centers had received ethical approval by the local institutional review board.

## Conflicts of Interest

The authors declare no conflicts of interest.

## Supporting information




**Table S1**: The risk of LN at 1‐, 3‐, 5‐ years based on GRS quartiles
**Table S2**: Definitions of the 18 candidate predictors included in this study.
**Figure S1**: The overall incidence of new‐onset LN in the discovery (A) and validation (B) cohort.
**Figure S2**: Variable selection using the least absolute shrinkage and selection 23 operator (LASSO). (A) LASSO model coefficient profiles of the 18 candidate variables. (B) Tuning parameter selection by cross‐validation in the LASSO model. The solid vertical lines represent the partial likelihood deviance standard error (SE). The red dotted line indicates the cross‐validation curve. The broken vertical lines indicate the optimal values on the basis of the minimum criteria and 1‐SE criteria.
**Figure S3**: Distributions of the density of GRS score and the number 29 of risk alleles by new‐onset LN. (A) the density of GRS in SLE patients developed and without LN during follow‐up. (B) the number of risk alleles in developed and without LN during follow‐up.

## Data Availability

The datasets analyzed during the current study are not publicly available due to the data also forming part of an ongoing study, but are available from the corresponding author on reasonable request.
