# Supplementary material for: Applying Genetic Risk Score to Improve Risk Assessment of New‐Onset Lupus Nephritis in Systemic Lupus Erythematosus: A Large‐Scale Prospective Cohort Study
Source: MedComm (2020). 2025 Nov 14;6(12):e70453. doi: 10.1002/mco2.70453 (PMC12618856; doi:10.1002/mco2.70453)
Supplement: Supplementary file 1 — Table S1: The risk of LN at 1‐, 3‐, 5‐ years based on GRS quartiles Table S2: Definitions of the 18 candidate predictors included in this study. Figure S1: The overall incidence of new‐onset LN in the discovery (A) and validation (B) cohort. Figure S2: Variable selection using the least absolute shrinkage and selection 23 operator (LASSO). (A) LASSO model coefficient profiles of the 18 candidate variables. (B) Tuning parameter selection by cross‐validation in the LASSO model. The solid vertical lines represent the partial likelihood deviance standard error (SE). The red dotted line indicates the cross‐validation curve. The broken vertical lines indicate the optimal values on the basis of the minimum criteria and 1‐SE criteria. Figure S3: Distributions of the density of GRS score and the number 29 of risk alleles by new‐onset LN. (A) the density of GRS in SLE patients developed and without LN during follow‐up. (B) the number of risk alleles in developed and without LN during follow‐up. [file MCO2-6-e70453-s001.pdf]

Applying genetic risk score to improve risk assessment of new-onset lupus nephritis in systemic lupus erythematosus: a large-scale prospective cohort (CSTAR) study

1. Results

We evaluated the incidence rate of LN at 1-, 3-, and 5-years based on GRS quartiles. As shown in Table S1, the 3-year cumulative incidence of LN in low-, medium-, and high-GRS quartiles were 3.0%, 8.7%, and 17.4%, respectively.

Table S1. The risk of LN at 1-, 3-, 5- years based on GRS quartiles

	Low-quartile<br><7.81 (N=113)	Median-quartile<br>7.52-8.13 (N=225)	High-quartile<br>>8.13 (N=113)
1-year	1.9%	5.3%	6.7%
3-years	3.0%	8.7%	17.4%
5-years	6.3%	11.6%	22.1%

2. Materials and Methods

In the risk factors analysis, candidate predictors for new-onset LN included age, gender, SLE disease duration, clinical manifestations, and autoantibody profiles. The definitions of the 18 candidate predictors are provided in Table S2.

Table S2. Definitions of the 18 candidate predictors included in this study.

Variables	Definitions
**General information**	
Age	Age at diagnosis of SLE.
Gender	Male or female.
SLE disease duration	Years from onset to diagnosis.
**Clinical manifestations**	
Malar rash	Malar rash observed by a clinician during the disease course.
Discoid skin lesions	Erythematous-violaceous cutaneous lesions with secondary changes of atrophic scarring, dyspigmentation, often follicular hyperkeratosis or plugging (scalp) during the disease course.
Arthritis	Synovitis involving two or more joints during the disease course, characterized by swelling or effusion or tenderness in 2 or more joints

	and thirty minutes or more of morning stiffness.
Oral ulcerations	Palate buccal, tongue or nasal ulcers during the disease course in the absence of other causes.
Alopecia	Diffuse thinning or hair fragility with visible broken hairs observed by a clinician during the disease course.
Serositis	Typical pleurisy, pleural effusions, or pleural rub during the disease course;<br>Typical pericardial pain, pericardial effusion or pericardial rub, or pericarditis by EKG during the disease course.
Neurological involvement	Seizures, psychosis, mononeuritis multiplex, vasculitis myelitis, peripheral or cranial neuropathy, history of cerebrovascular accidents, or acute confusional state during the disease course in the absence of offending drugs or known metabolic derangements.
Hematological involvement	Hemolytic anemia with reticulocytosis or leukopenia ($<4,000/mm^3$ on ≥ 2 occasions) or lymphopenia ($<1,500/mm^3$ on ≥ 2 occasions) or thrombocytopenia ($<100,000/mm^3$) during the disease course in the absence of offending drugs.
**Laboratory features**	
Hypocomplementemia	Low C3 and (or) low C4 at SLE diagnosis.
**Autoantibodies profile**	
Anti-dsDNA antibodies	Tested by IIF using Crithidia luciliae immunofluorescence test (CLIFT) and enzyme-linked immunosorbent assay (ELISA). Either CLIFT or ELISA showed positive result at SLE diagnosis was defined as positive anti-dsDNA antibodies.
Anti-Sm antibodies<br>Anti-RNP antibodies<br>Anti-SSA antibodies<br>Anti-SSB antibodies<br>Anti-rRNP antibodies	The immunoblotting assay using the EUROLINE ENA Profile 14 Ag (Euroimmun) according to the manufacturer's instructions.<br>The autoantibodies profile was assessed at SLE diagnosis and during follow-ups.

Supplementary Figure

A

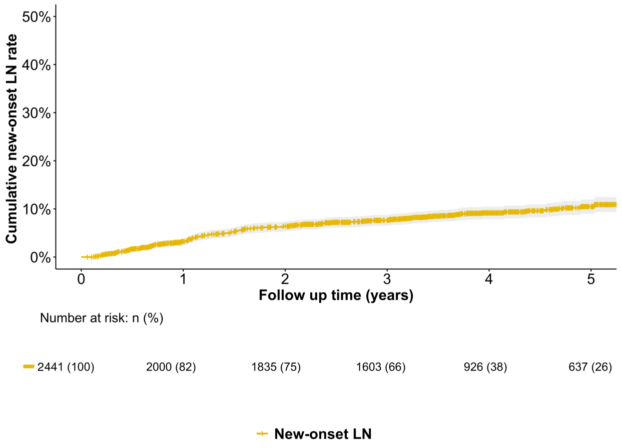

B

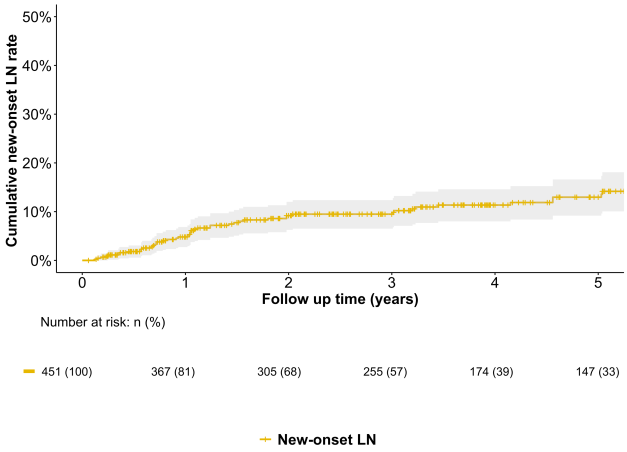

Figure S1. The overall incidence of new-onset LN in the discovery (A) and validation (B) cohort.

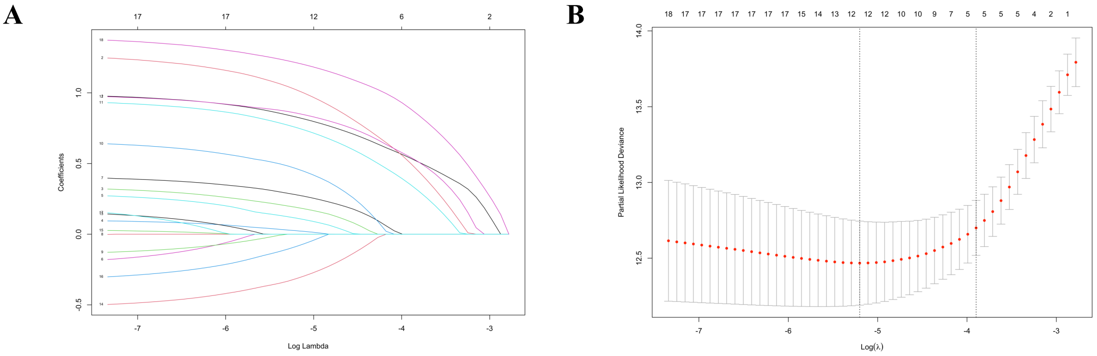

Figure S2. Variable selection using the least absolute shrinkage and selection operator (LASSO). (A) LASSO model coefficient profiles of the 18 candidate variables. (B) Tuning parameter selection by cross-validation in the LASSO model. The solid vertical lines represent the partial likelihood deviance standard error (SE). The red dotted line indicates the cross-validation curve. The broken vertical lines indicate the optimal values on the basis of the minimum criteria and 1−SE criteria.

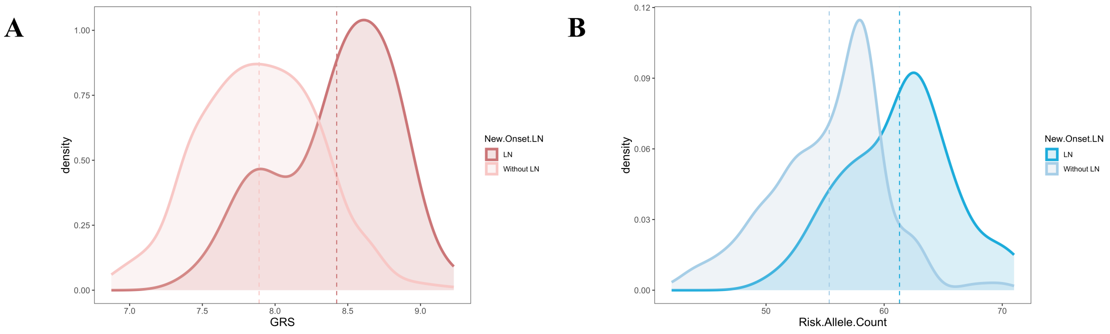

Figure S3. Distributions of the density of GRS score and the number of risk alleles by new-onset LN. (A) the density of GRS in SLE patients developed and without LN during follow-up. (B) the number of risk alleles in developed and without LN during follow-up.
